# Supplementary material for: Heat adaptation of phage T7 under an extended genetic code
Source: Virus Evol. 2021 Dec 1;7(2):veab100. doi: 10.1093/ve/veab100 (PMC8923235; doi:10.1093/ve/veab100)
Supplement: veab100_Supp [file veab100_supp.zip › Sequencing_Analysis.zip!/Sequencing_Analysis/A1/evidence/DEL_16.html]

BRESEQ :: Evidence


| Predicted mutation | | | | | | |
| --- | --- | --- | --- | --- | --- | --- |
| evidence | seq id | position | mutation | annotation | gene | description |
| RA | NC\_001604.1 | 39,815 | (G)_5→4_ | intergenic (–/–) | – / – | –/– |

| Read alignment evidence... | | | | | | | | | | | |
| --- | --- | --- | --- | --- | --- | --- | --- | --- | --- | --- | --- |
|  | seq id | position | | ref | new | freq | score (cons/poly) | reads | annotation | genes | product |
| \* | NC\_001604.1 | 39,811 | 0 | G | . | 95.2% | 3138.7 / 197.2 | 912 | intergenic (–/–) | *–/–* | –/– |
| Reads supporting (aligned to +/- strand):  **ref** base G (17/26);  **new** base . (343/508);  **total** (375/537) | | | | | | | | | | | |
| Fisher's exact test for biased strand distribution *p*-value = 1.00e+00 | | | | | | | | | | | |
| Kolmogorov-Smirnov test that lower quality scores support variant *p*-value = 9.62e-01 | | | | | | | | | | | |

Only 100 of 1007 total aligned reads displayed.  

`TTGCTCTATACTCACTTGTAGCGATTAGGGTCTTCCTGACCGACTGATGGCTCACCGAGGGATTCAGCGGTATGATTGCATCACACCACTTCATCCCTATAGAGTCAAGTCCTAAGGTATACCCATAA‑AGAGCCTCTAATGGTCTATCCTAAGGTCTATACCT‑AAAGATAGGCCATCCTATCAGTGTCACCTAAAGAGGGTCTTAGAGAGGGCCTATGGAGTTCCTATAGGGTCCTTTAAAATATACCATAAAAATCTGAGTGACTATCTCACAGTGTACGGACCTAAAGTTCCCCCATAGGGGGTACCTAAAGCCCAGCCAATCACCTAAAGTCAACCTTCGGTTGACCTTGAGGGTTCCCTAAGGGTTGGGGATGACCCTTGGGTTTGTCTTTGGGTGTTACCTTGAGTGTCTCTCTGTGTCCCT‑‑‑‑‑‑‑‑‑‑  >  NC_001604.1/39511‑39937`  

                                                                                                                                                                                                                                                                                                              `|`                                                                                                                                          
`tttctttataCTCACTTGTAGCGATTAGGGTCTTCCTGACCGACTGATGGCTCACCGAGGGATTCAGCGGTATGATTGCATCACACCACTTCATCCCTATAGAGTCAAGTCCTAAGGTATACCCATAA‑AGAGCCTCTAATGGTCTATCCTAAGGTCTATACCT‑AAAGATAGGCCATCCTATCAGTGTCACCTAAAGAGGGTCTTAGAGAGGGCCTATGGAGTTCCTATAGGGTCCTTTAAAATATACCATAAAAATCTGAGTGACTATCTCACAGTGTACGGACCTAAAGTTCCCACATAg                                                                                                                                          <  1:321447/295‑1 (MQ=255)`  
 `gattaaggcacagtttggagctgatAGGATCTTCCTAACCGACTGATGGCTAAACGAGGGATTCAGGGGTATGATTGCATCACACCACTTCAGCCCTATAAAGTCAAGTTCTAAGGTATACCCATAA‑AGAGCCTCTAATGGGCTATCCTAAGGTCTATACCT‑AAAGATAGGCCAGCCGATCAGTGTCACCTAAAGAGGGTCTTAGAGAGGGCCTATGGAGTTCCTATAGGGTCCTTTAAAATATACCATAAAAATCTGAGTGACTATCTCACAGTGTACGGACCTAAATTTCCCCCATAgg                                                                                                                                         <  1:490262/277‑1 (MQ=255)`  
  `gCTCTATACTCACTTGTAGCGATTAGGGTCTTCCTGACCGACTGATGGCTCACCGAGGGATTCAGCGGTATGATTGCATCACACCACTTCATCCCTATAGAGTCAAGTCCTAAGGTATACCCATAA‑AGATCCTCTAATGGTCTATCCTAAGGTCTATACCT‑AAAGATAGGCCATCCTATCAGTGTCACCTAAAGAGGGTCTTAGAGAGGGCCTATGGAGTTCCTATAGGGTCCTTTAAAATATACCATAAAAATCTGAGTGACTATCTCACAGTTTACGGACCTAACGTTCCCCCATAggg                                                                                                                                        >  1:387929/1‑301 (MQ=255)`  
     `ctATACTCACTTGTAGCGATTAGGGTCTTCCTGACCGACTGATGGCTCACCGAGGGATTCAGCGGTATGATTGCATCACACCACTTCATCCCTATAGAGTCAAGTCCTAAGGTATACCCATAA‑AGAGCCTCTAATGGTCTATCCTAAGGTCTATACCT‑AAAGATAGGCCATCCTATCAGTGTCACCTAAAGAGGGTCTTAGAGAGGGCCTATGGAGTTCCTATAGGGTCCTTTAAAATATACCATAAAAATCTGAGTGACTATCTCACAGTGTACGGACCTAAAGTTCCCCCATAggggta                                                                                                                                     >  1:74683/1‑299 (MQ=255)`  
     `ctATACTCACTTGTAGCGATTAGGGTCTTCCTGACCGACTGATGGCTCACCGAGGGATTCAGCGGTATGATTGCATCACACCACTTCATCCCTATAGAGTCAAGTCCTAAGGTATACCCATAA‑AGAGCCTCTAATGGTCTATCCTAAGGTCTATACCT‑AAAGATAGGCCATCCTATCAGTGTCACCTAAAGAGGGTCTTAGAGAGGGCCTATGGAGTTCCTACAGGGTCCTTTAAAATATACCATAAAAATCTGAGTGACTATCTCACAGTGTACGGACCTAAAGTTCCCCCATAggggtc                                                                                                                                     >  1:410910/1‑299 (MQ=255)`  
       `ataCTCACTTTTAGCGATTAGGGTCTTCCTGACCGACTGATGGCTCACCGAGGGATTCAGCGGTATGATTGCATCACACCACTTCATCCCTATAGAGTCAAGTCCTAAGGTATACCCATAA‑AGAGCCTCTAATGGTCTATCCTAAGGTCTATACCT‑AAAGATAGGCCATCCTATCAGTGTCACCTAAAGAGGGTCTTAGAGAGGGCCTATGGAGTTCCTATAGGGTCCTTTAAAATATACCATAAAAATCTGAGTGACTATCTCACAGTGTACGGACCTAAAGTTCCACCATAggggtacc                                                                                                                                   <  1:91265/301‑5 (MQ=255)`  
       `ataCTCACTTGTAGCGATTAGGGTCTTCCTGACCGACTGATGGCTCACCGAGGGATTCAGCGGTATGATTGCATCACACCACTTCATCCCTATAGAGTCAAGTCCTAAGGTATACCCATAA‑AGAGCCTCTAATGGTCTATCCTAAGGTCTATACCT‑AAAGATAGGCCATCCTATCAGTGTCACCTAAAGAGGGTATTAGAGAAGGCCTATGGAGTTCCTATAGGGTCCTTTAAAATCTACCATAAAAATCTGAGTGACTATCTCACAGTGTACGAACCTAAAGTTCCCCCATAggggtacc                                                                                                                                   >  1:475531/1‑297 (MQ=255)`  
       `ataCTCACTTGTAGCGATTAGGGTCTTCCTGACCGACTGATGGCTCACCGAGGGATTCAGCGGTATGATTGCATCACACCACTTCATCCCTATAGAGTCAAGTCCTAAGGTATACCCATAA‑AGAGCCTCTAATGGTCTATCCTAAGGTATATACCT‑ACAGATAGGCCATCCTATCAGTGTCACCTAAAGAGGGTCTTAGAGAGGGCCTATGGAGTTCCTCTAGGGTCCTTTAAAATATACCATAAACATCTGAGTGACTATATCCCAGTGTACGGACCTAAAGTTCCCCCATAggggtacc                                                                                                                                   >  1:146300/1‑297 (MQ=255)`  
       `ataCTCACTTGTAGCGATTAGGGTCTTCCTGACCGACTGATGGCTCACCGAGGGATTCAGCGGTATGATTGCATCACACCACTTCATCCCTATAGAGTCAAGTCCTAAGGTATACCCATAA‑AGAGCCTCTAATGGTCTATCCCAAGGTCTATACCT‑AAAGATAGGCCATCCTATCAGTGTCACCTAAAGAGGGTCTTAGAGAGGGCCTATGGAGTTCCTATAGGGTCCTTTAAAATATACCATAAAAATCTGAGTGACTATCTCACAGTGTACGGACCTAAAGTTCCCCCATAggggtacc                                                                                                                                   >  1:463424/1‑297 (MQ=255)`  
       `ataCTCACTTGTAGCGATTAGGGTCTTCCTGACCGACTGATGGCTCACCGAGGGAATCAGCGGAATGATTGCATCACACCACTTCATCCCTATAGAGTCAAGTCCTAAGGTATACCCATAA‑AGAGCCTCTAATGGTCTATCCTAAGGTCTATACCT‑AAAGATAGGCCATCCTATCAGTGTCACCTAAAGAGGGTCTTAGAGAGGGCCTATGGAGTTCCTATAGGGTCCTTTAAAATATACCATAAAAATCTGAGTGACTATCTCACATTGTACGGACCTAAAGTTCCCCCATAggggtacc                                                                                                                                   >  1:365855/1‑297 (MQ=255)`  
        `taCTCACTTGTAGCGATTAGGGTCTTCCTGACCGACTGATGGCTCACCGAGGGATTCAGCGGTATGATTGCATCACACCACTTCATCCCTATAGAGTCAAGTCCTAAGGTATACCCATAA‑AGAGCCTCTAATGGTCTATCCTAAGGTCTATACCT‑AAAGATAGTCCATCCTATCAGTGTCACCTAAAGAGGGTCTTAGAGAGGGCCTATGGAGTTCCTATAGGGTCCTTTAAAATATACCATAAAAATCTGAGTGACTATCTCACAGTGTACGGACCTAAAGTTCCCCCATA‑GGGGTACCt                                                                                                                                 <  1:23514/301‑1 (MQ=255)`  
         `aCTCACTTGTAGCTATTAGGGTCTTCCTGACCGACTGATGGCTCACCGAGGGATTCAGCGGTATGATTGCATCACACCACTTCATCCCTATAGAGTCAAGTCCTAAGGTATACCCATAA‑AGAGCCTCTAATGGTCTATCCTAAGGTCTATACCT‑AAAGATAGGCCATCCTATCAGTGTCACCTAAAGAGGGTCTTAGAGAGGGCCTATGGAGTTCCTATAGGGTCCTTTAAAATATACCATAAAAATCTGAGTGACTATCTCACAGTGTACGGACCTAAAGTTCCCCCATA‑GGGGTACCTa                                                                                                                                <  1:395188/301‑1 (MQ=255)`  
            `cACTTGTAGCGATTATGGTCTTCCTGACCGACTGATGGCTCACCGCGGGATTCAGCGGTATGATTGCATCACACCACTTCATCCCTCTAGAGTCCAGTCCTAAGGTATACCAATAA‑AGAGCCTCTAATGGTCTATCCTAAGGTCTATACAT‑AAAGATACACCATCCTATCAGTGTCACCTAAAGAGGGTCTTAGTGAGGGCCTATGGAGTTACTATAGGGTCCTTTAAAATATACCATAAAAATCTGATTGACTCTCCCCCAGTGTACTGACCTAAAGTTCCCCCCCA‑GGGGTCCCTaaac                                                                                                                             >  1:196771/1‑300 (MQ=255)`  
            `cACTTGTAGCGATTAGGGTCTTCATGACCGACTGATGGCTCACCGAGGGATTCAGCGGTATGATTGCATCACACCACTTCATCCCTATAGAGTCAAGTACTAAGGTATACCCATAA‑AGAGCCTCTAATGGTCTATCCTAAGGTCTATACCT‑AAAGATAGGCCATCCTATCAGTGTCACCTAAAGAGGGTCTTAGAGAGGGCCTATGGAGTTCCTATAGGGTCCTTTAAAATATACCATAAAAATCTGAGTGACTATCTCACAGTGTACGGACCTAAAGTT‑CCCCATAggggtctgtctctt                                                                                                                             >  1:189559/1‑291 (MQ=255)`  
             `aatgtttatatgagacagcttcttGACCGACTGATTGCTCACCGAGGTATTAAGTGGTATGATTACATCACACCACTTCAGTCCTATAGAGTCAAGTCCTATGGTATACCCATAA‑ACATCCTCTAATGGTCTATCCTAAGGTCTATACCT‑AAAGCTAGGCCATCCTATCAGTGTCACCTAAAGAGGCTCTTAGAGAGGGCCTATGGAGTTCCTATAGGGTCCTTTAAAATATACCATAAAAATCTGAGTGACTATCTCACAGTGTACGGACCTAAAGTTCCCCCATA‑GGGGTACCTAAAGc                                                                                                                            <  1:80772/283‑1 (MQ=255)`  
             `aCTTGTAGCGATTAGGGTCTTCCTGACCGACTGATGGCTCACCGAGGGATTCAGCGGTATGATTGCATCACACCACTTCATCCCTATAGAGTCAAGTCCTAAGGTATACACATAA‑AGAGCCTCTAATGGTCTATCCTAAGGTCTATACCT‑AAAGATAGGCCATCCTATCAGTGTCACCTAAAGAGGGTCTTAGAGAGGGCCTATGGAGTTCCTGTAGGGTCCTTTAAAATATACCATAAAAACCTGAGTGACTATCTCACAGTGTACGGACCTAAAGTTCCCCCATA‑GGGGTACCTAAAGc                                                                                                                            <  1:163624/301‑1 (MQ=255)`  
                 `gTAGCGATTAGGGTCTTCCTGACCGACTGATGGCTCACCGAGGGATTCAGCGGTATGATTGCATCACACCACTTCATCCCTATAGAGTCAAGTCCTAAGGTATACCCATAA‑AGAGCCTCTAATGGTCTATCCTAAGGTCTATACCTAAAAGATAGGCCATCCTATCAGTGTCACCTAAAGAGGGTCTTAGAGAGGGCCTATGGAGTTCCTATAGGGTCCTTTAAAATATACCATAAAAATCTGAGTGACTATCTCACAGTGTACGGACCTAAAGTTCCCCCATAGGGGGTACCTAAAGcgc                                                                                                                          >  1:2205/1‑299 (MQ=255)`  
                   `agtgATTAGGGTCATCCTGACCGACTGATGGCTCACCGAGGGATTCAGCGGTATGATTGCATCAAACCATTTCATCCCTATAGAGTCAATTCCTAAGGTATACCCATAA‑AGAGCCTCTAATGGTCTATACTAAGGTCTATACCT‑AAAGAGAGGCCATCCTATCAGTGTATCCTAAAGAGGGTCTTAGAGAGGGCCTATGGAGTTCCTATAGGGTCCTTTAAAATATACCATAAAAATCTGAGTGACTATCTCACAGTGTACGGACCTAAAGTTCCCCCATA‑GGGGTACCTAAAGCCCAGcc                                                                                                                      <  1:431480/298‑1 (MQ=255)`  
                         `tAGGGTCTTCCTGACCGACTGATGGCTCACCGAGGGATTCAGCGGTATGAGTGCATCACACCACTTCATCCCTATAGAGTCAAGTCCTAAGGTATACCCATAA‑AGAGCCTCTAATGGTCTATCCTAAGGTCTATACCT‑AAAGATAGGCCATCCTATCAGTGTCACCTAAAGAGGGTCTTAGAGAGGGCCTATGGAGTTCCTATAGGGTCCTTTAAAATATACCATAAAAATCTGAGTGACTATCTCACAGTGTACGGACCTAAAGTTCCCCCATA‑GGGGTACCTAAAGCCCAGCCAATCAc                                                                                                                <  1:293781/301‑1 (MQ=255)`  
                          `agatgtgtataagagacaggATGGCTCACCGAGGGATTCAGCGGTATGATTGCATCACACCACTTCATCCCTATAGAGTCAATTCCTAAGGTATACCCATAA‑AGAGCCTCTAATGGTCTATCCTAAGGTCTATACCT‑AAAGATAGGCCATCCTATCAGTGTCACCTAAAGAGGGTCTTAGAGAGGGCCTATGGAGTTCCTATAGGGTCCTTTAAAATATACCATAAAAATCTGAGTGACTATCTCACAGTGTACGGACCTAAAGTTCCCCCATA‑GGGGTACCTAAAGCCCAGCCAATCAcc                                                                                                               <  1:148207/282‑1 (MQ=255)`  
                           `gggTCTTCCTGACCGACTGATGGCTCACCGAGGGATTCAGCGGTATGATTGCATCACACCACTTCATCCCTATAGAGTCAAGTACTAAGGTATACCCATAA‑AGAGCCTCTAATGGTCTATCCTAAGGTATATACCT‑AAAGATAGGCCATCCTATCCATGTCACCTAAAGAGGGTCTTAGAGATGGCCTATGGAGTTCCTATAGGGTCCTTTAAAATATACCATAAAAATCTGAGTGACTATCTCACAGTGTACGGCCCTAAAGTTCCCACATA‑GGGGTACCTAAAACCCAGCCAATCACCt                                                                                                              >  1:193785/1‑301 (MQ=255)`  
                               `cTTCCTGACCGACTGATGGCTCACCGAGGGATTCAGCGGTATGATTGCATCACACCACTTCATCCCTATAGAGTCAAGTCCTAAGGTATACCCATAA‑AGAGCCTCTAATGGTCTATCCTAAGGTCTATACCT‑AAAGATAGGCCATCCTCTCAGTGTCACCTAACGAGGGTCTTAGAGAGGGCCTATGGAGTTCCTATAGGGTCCTTTAAAATATACCATAAAAATCTGAGTGACTATCTCACAGTGTACGGACCTAAAGTTTCCCCATA‑GGGGTACATAAAACCCAGACACTCACCTaaaa                                                                                                          >  1:546/1‑300 (MQ=255)`  
                               `cTTCCTGACCGACTGATGGCTCACCGAGGGATTCAGCGGTATGATTGCATCACACCACTTCATCCCTATAGAGTCAAGTCCTAAGGTATACCCATAA‑AGAGCCTCTAATGGTCTATCCTAAGGTCTATACCT‑AAAGATAGGCCATCCTATCAGTGTCACCTAAAGAGGGTCTTAGAGAGGGCCTATGGAGTTCCTATAGGGTCCTTTAAACTATACCATAAAAATCTAAGTGACTATCTCACAGTGTACGGACCTAAAGTTCCCCCATA‑GGGGTACCTACAGCCCAGCCAATCACCTAAAg                                                                                                          >  1:340206/1‑301 (MQ=255)`  
                               `cTTCCTGACCGACTGATGGCTCACCGAGGGATTCAGCGGTATGATTGCATCACACCACTTCATCCCTATAGAGTCAAGTCCTAAGGTATACCCATAA‑AGAGCCTCTAATGGTCTATCCTAAGGTCTATACCT‑AAAGATAGGCCATCCTATCAGTGTCACCTAAAGAGGGTCTTAGAGAGGGCCTATGGAGTTCCTATAGGGTCCTTTAAAATATACCATAAAAATCTGAGTGACTATCTCACAGTGTACGGACCTAAAGTTCCCCCATA‑GGGGTACCTAAAGCCCAGCCAATCACCTAAAg                                                                                                          <  1:418649/301‑1 (MQ=255)`  
                               `cTTCCTGACCGACTGATGGCTCACCGAGGGATTCAGCGGTATGATTGCATCACACCACTTCATCCCTATAGAGTCAAGTCCTAAGGTATACCCATAA‑AGAGCCTCTAATGGTCTATCCTAAGGTCTATACCT‑AAAGATAGGCCATCCTATCAGTGTCACCTAAAGAGGGTCTTAGAGAGGGCCTATGGAGTTCCTATAGGGTCCTTTAAAATATACCATAAAAATCTGAGTGACTATCTCACAGTGTACGGACCTAAAGTTCCCCCATA‑GGGGTACCTAAAGACCAGCCAATCACCTAAAg                                                                                                          >  1:398432/1‑301 (MQ=255)`  
                               `cTTCCTGACCGACTGATGGCTCACCGAGGGATTCAGCGGTATGATTGCATCACACCACTTCATCCCTATAGAGTCAAGTCCTAAGGTATACCCATAA‑AGAGCCTCTAATGGTCTATCCTAAGGTCTATACCT‑AAAGATAGGCCATCCTATCAGTGTCACCTAAAGAGGGTCTTAGAGAGGGCCTATGGAGTTCCTATAGGGTCCTTTAAAATATACAATAAAAATCTGAGTGACTATCTAACCATGTACGGACCTAAAGTTCCCCCATA‑GGTGTACCTAAAGCCCAGCCAATCACCTAAAg                                                                                                          >  1:94593/1‑301 (MQ=255)`  
                                       `atctttaggtatagaccttaggATTCAGCGGTATGATTACATCACACCACTTCATCCCTATAGAGTCAAGTCCTAAGGTAAACCCATAA‑AGAGCCTCTAATGGTCTATCCTAAGGTCTATACCT‑AAAGATAGGCCATCCTATCAGTGTCACCTAAAGAGGGTCTTAGAGAGGGCCTATGGAGTTCCTATAGGGTCCTTTAAAATATACCATAAAAATCTGAGTGACTATCTCACAGTGTACGGCCCTAAAGTTACCCCATA‑GGGGTACCTAAAGCCAAGCCAATCACCTAAAgctgtctct                                                                                                  >  1:204356/21‑293 (MQ=255)`  
                                          `aCTGATGGCTCACCGAGGGATTCAGCGGTATGATTGCATCACACCACTTCATCCCTATAGAGTCAAGTCCTAAGGTATACCCATAA‑AGAGCCTCTAATGGTCTATCCTAAGGTCTATACCT‑AAAGATAGGCCATCCTATCAGTGTCACCTAAAGAGGGTCTTAGAGAGGGCCTATGGAGTTCCTATAGGGTCCTTTAAAATATACCATAAAAATCTGAGTGACTATCTCACAGTGTACGGACCTAAAGTTCCCCCATA‑GGGGTACCTAAATCCCAGCCAATCACCTAAAGTCAACCTTCgg                                                                                               >  1:405418/1‑301 (MQ=255)`  
                                          `aCTGATGGCTCACCGAGGGATTCAGCGGTATGATTGCATCACACCACTTCATCCCTATAGAGTCAAGTCCTAAGGTATACCCATAA‑AGAGCCTCTAATGGTCTATCCTAAGGTCTATACCT‑AAAGATAGGCCATCATATCAGTGTCACCTAAAGAGGGTCTTAGAGAGGGCCTATGGAGTTCCTATAGGGTCCTTTAAAATATACCATAAAAATCTGAGTGACTATCTCACAGTGTACGGACCTAAAGTTCCCCCATA‑GGGGTACCTAAAGCCCAGCCACTcctgtctcttatacacatct                                                                                               >  1:6588/1‑282 (MQ=255)`  
                                             `aaTGACTAACCGAGGTGTTCAGCGGAATGAATGCATCACACCACTGCATCCATATAGAGTAAAGGCCAAAGGTATACCCATAA‑AGAGCCTCTAATGATCTATCCTAAGGTCTATACCT‑AAAGATAGGCAATCATATCAGTGTCACCTAAAGAGGGTCTTAGAGAGGGCCTATGGAGTTCCTATAGGGTCCTTTAAAATATACCATAAAAATCTGAGTGACTATCTCACAGTGTACGGACCTAAAGTTCCCCCATA‑GGGGTACCTAAAGCCCAGCCAATCACCTAAAGTCAACCTTAGTTTg                                                                                            <  1:368210/300‑1 (MQ=38)`  
                                                 `gatCACCGAGGGATTCAGCGGTATGATTGTATCACACCACTTCATCCCTATAGAGTCAAGTCCTAAGGTATACCCATAA‑AGAGCCTCTAATGGTCTATCCTAAGGTCTATACCT‑AAAGATAGGCCATCCTATCAGTGTCACCTAAAGAGTGTCTTAGAGAGGGCCTATGGAGTTCCTATAGGGTCCTTTAAAATATACCATAAAAATCTGAGTGACTATCTCACAGTGTACGGACCTAAAGTTCCCCCATA‑GGGGTACCTAAAGCCCAGCCAATCACCTAAAGTCAACCTTCGGTTGACCt                                                                                        <  1:497106/299‑1 (MQ=255)`  
                                                  `cTCACCGAGGGATTCAGCGGTATGATTGCATCACACCACTTCATCCCTATAGAGTCAAGTCCTAAGGTATACCCATAA‑AGAGCCTCTAATGGTCTATCCTAAGGTCTATACCT‑AAAGATAGGCCATCCTATCAGTGTCACCTAAAGAGGGTCTTAGAGAGGGCCTATGGAGTTCCTATAGGGTCCTTTAAAATATACCATAAAAATCTGAGTGACTATCTCACAGTGTACGGACCTAAAGTTCCCCCATA‑GGGGTACCTAAAGCCCAGCCAATCACCTAAAGTCAACCTTCGGTTGACCtt                                                                                       <  1:132630/301‑1 (MQ=255)`  
                                                      `ccGAGGGATTCAGCGGTATGATTGCATCACACCACTTCATCCCTATAGAGTCAAGTCCTAAGGTATACCCATAA‑AGAGCCTCTAATGGTCTATCCTAAGGTCTATACCT‑AAAGATAGGCCATCCTATCAGTGTCACCTAAAGAGGGTCTTAGAGAGGGCCTATGGAGTTCCTATAGGGTCCTTTAAAATATACCATAAAAATCTGAGTGACTATCTCACAGTGTACGGACCTAAAGTTCCCCCATA‑GGGGTACCTAAAGCCCAGCCAATCACCTAAAGTCAACCTTCGGTTGACCTTGAgg                                                                                   <  1:268184/301‑1 (MQ=255)`  
                                                       `cGAGGGATACAGCGGTATGATTGCATCAAACCACGTCATCCCTATAGAGTAAAGTCCTAAGGTATACCCATAA‑AGAGCCTATAATGGTCTATCCTAAGGTCTATACCT‑AAAGATAGGCCATCCTATCAGTGTCACCTAAAGAGGGTCTTAGAGAGGGCCTATGGAGTTCCTATATGGTCCTTTAAAATATACCATAAAAATCTGAGTGACTATCTCACAGTGTACGGACCTAAAGTTCCCCCATA‑GGGGTACCTAAAGCCCAGCCAATCACCTAAAGTCAACCTTCGGTTGACCTTGAggg                                                                                  <  1:342335/301‑1 (MQ=255)`  
                                                           `ggATTCAGCGGTATGATTGCATCACACCACTTCATCCCTATAGAGTCAAGTCCTAAGGTATACCCATAA‑AGAGCCTCTAATGGTCTATCCTAAGGTCTATACCT‑AAAGATAGGCCATCCTATCAGTGTCACCTAAAGAGGGTCTTAGAGAGGGCCTATGGAGTTCCTATCGGGTCCTTTAAAATATACCATAAAAATCTGAGTGACTATCTCACAGTGTACGGACCTAAAGTTCCCCCATA‑GGGGTACCTAAAGCCCAGCCAATCACCTAAAgctgtctcttatacacatctccgagcccc                                                                              >  1:24209/1‑273 (MQ=255)`  
                                                           `ggATTCAGCGGTATGATTGCATCACACCACTTCATCCCTATAGAGTCAAGTCCTAAGGTATACCCATAA‑AGAGCCTCTAATGGTCTATCCTAAGGTCTATACCT‑AAAGATAGGCCATCCTATCAGTGTCACCTAAAGAGGGTCTTAGAGAGGGCCTATGGAGTTCCTATAGGGTCCTTTAAAATATACCATAAAAATCTGAGTGACTATCTCACAGTGTACGGACCTAAAGTTCCCCCATA‑GGGGTACCTAAAGCCCAGCCAATCACCTAAAGTCAACCTTCGGTTGACCTTGAGGGTTct                                                                              >  1:358382/1‑300 (MQ=255)`  
                                                           `ggATTCAGCGGTATGATTGCATCACACCACTTCATCCCTATAGAGTCAAGTCCTAAGGTATACCCATAA‑AGAGCCTCTAATGGTCTATCCTAAGGTCTATACCT‑AAAGATAGGCCATCCTATCAGTGTCACCTAAAGAGGGTCTTAGAGAGGGCCTATGGAGTTCCTATAGGGTCCTTTAAAATATACCATAAAAATCTGAGTGACTATCTCACAGTGTACGGACCTAAAGTTCCCCCATA‑GGGGTACCTAAAGCCCAGCCAATCACCTAAAGTCAACCTTCGGTTGACCTTGAGGGTTcc                                                                              >  1:362899/1‑301 (MQ=255)`  
                                                           `ggATTCAGCGGTATGATTGCATCACACCACTTCATCCCTATAGAGTCAAGTCCTAAGGTATACCCATAA‑AGAGCCTCTAATGGTCTATCCTAAGGTCTATACCT‑AAAGATAGGCCATCCTATCAGTGTCACCTAAAGAGGGTCTTAGAGAGGGCCTATGGAGTTCCTATAGGGTCCTTTAAAATATACCATAAAAATCTGAGTGACTATCTCACAGTCTACGTACCTAAAGTTCCCCC‑TTGCTGGTACCTAACGCCCAGCCAATCCCCTAAAGCCACCTTTCGGTGTACCttatggctttc                                                                              >  1:468890/1‑292 (MQ=255)`  
                                                               `gtgtataagagacagCATCACACCACTTCATCCCTATAGAGTCAAGTCCTAAGGTATACCCATAA‑AGAGCCTCTAATGGTCTATCCTAAGGTCTATACCT‑AAAGATAGGCCATCCTATCAGTGTCACCTAAAGAGGGTCTTAGAGAGGGCCTATGGAGTTCCTATAGGGTCCTTTAAAATATACCATAAAAATCTGAGTGACTATCTCACAGTGTACGGACCTAAAGTTCCCCCATA‑GGGGTACCTAAAGCCCAGCCAATCACCTAAAGTCAACCTTCGGTTGACCTTGAGGGTTCCCTaa                                                                          <  1:88094/287‑1 (MQ=255)`  
                                                                  `ggggTATGATTGAATCACACCACTTCATCCCTAAAGAGTCAAGTCCTAAGGTATACCCATAA‑AGAGCCTCTAATGGTCTATCCTAAGGTCTATACCT‑AAAGATAGGCCATCCTATCAGTGTCACCTAAAGGGGGTCTTAGAGAGGGCCTATGGAGTTCCTATAGGGTCCTTTAAAATATAACATAAAAATCTGAGTGACTATCTCACAGTGTACGGACCTAAAGTTCCCCCATA‑GGGGTACCTAAAGCCCAGCCAATCACCTAAAGTCAACCTTCGGTTGACCTTGAGGGTTCCCTAAggg                                                                       <  1:31660/299‑1 (MQ=255)`  
                                                                  `gcagTATGAATGAATCAAGCCACTGAATCCCTATAGAGTGAAGTCCTAAGGTATACCCATAA‑AGAGCCTCTAATGGTCTATCCTAAGGTCTATACAT‑AAAGATAGGCCATCCTATCAGTGTCACCTAAAGAGGGTCTTAGAGAGGGCCTAGGGAGTTGCTATAGGGTCATTTAAAATATACCATAAAAATCTGAGTGACTATCTCACAGTGTAAGGACCTAAAGTTCCCCCATA‑GGGGTACCTAAAGCCCAGCCAATCGCCTAAAGTCAACCTTCGGTTGACCTTGAGGGTTCCCTAAggg                                                                       <  1:380731/298‑1 (MQ=38)`  
                                                                  `gCGGTATGATTGCATCACACCACTTCATCCCTATAGAGTCAAGTCTTAAGGTATACCCATAA‑AGAGCCTCTAATGGTCTATCCTAAGGTCTATACCT‑AAAGATAGGCCATCCTATCAGTGTCACCTAAAGAGGGTCTTAGAGAGGGCCTATGGAGTTCCTATAGGGTCCTTTAAAATATACCATAAAAATCTGAGTGACTATCTCACAGTGTACGGACCTAAAGTTCCCCCATA‑GGGGTACCTAAAGCCCAGCCAATCACCTAAAGTCAACCTTCGGTTGACCTTGAGGGTTCCCTAAggg                                                                       <  1:22549/301‑1 (MQ=255)`  
                                                                  `atgtgtataagagacagcactacTTCATCCCTATAGAGTCAAGTCCTAAGGTATACCCATAA‑AGAGCCTCTAATGGTCTATCCTAAGGTCTATACCT‑AAAGATAGGCCATCCTATCAGTGTCACCTAAAGAGGGTCTTAGAGAGGGCCTATGGAGTTCCTATAGGGTCCTTTAAAATATACCATAAAAATCTGAGTGACTATCTCACAGTGTACGGACCTAAAGTTCCCCCATA‑GGGGTACCTAAAGCCCAGCCAATCACCTAAAGTCAACCTTCGGTTGACCTTGAGGGTTCCCTAAggg                                                                       <  1:157960/284‑1 (MQ=255)`  
                                                                   `cGGTATGATTGCATCACACCACTGCATCCCTATAGAGTCAAGTACTAAGGTATACCCATAA‑AGAGCCTCTAATGGTCTATCCTAAGGTCTATACCT‑AAAGATAGGCCATCCTATCAGTGTCACCTAAAGAGGGTCTTAGAGAGGGCCTATGGAGTTCCTATAGGGTCCTTTAAAATATACCATAAAAATCTGAGTGACTATCTCACAGTGTACGGACCTAAAGTTCACCCATA‑GGGGTACCTAAAGCCCAGCCAATCACCTAAAGTCAACCTTCGGTTGACCTTGAGGGTTCCCTAAGGGt                                                                      <  1:10476/301‑1 (MQ=255)`  
                                                                         `tataagagacagccacTTCATCCCTATAGAGTCAAGTCCTAAGGTATACCCATAA‑AGAGCCTCTAATGGTCTATCCTAAGGTCTATACCT‑AAAGATAGGCCATCCTATCAGTGTCACCTAAAGAGGGTCTTAGAGAGGGCCTATGGAGTTCCTATAGGGTCCTTTAAAATATACCATAAAAATCTGAGTGACTATCTCACAGTGTACGGACCTAAAGTTCCCCCATA‑GGGGTACCTAAAGCCCAGCCAATCACCTAAAGTCAACCTTCGGTTGACCTTGAGGGTTCCCTAAGGGTTGGGGa                                                                <  1:420896/289‑1 (MQ=255)`  
                                                                         `taTTGCATCACACCACTTCATCCCTATAGAGTTAAGTCCTAAGGTAAACCCATAA‑AGAGCCTCTAATGGTCTATCCTAAGGTCTATACCT‑AAAGATAGGCCATCCTATCAGTGTCACCTAAAGAGGGTCTTAGAGAGGGCCTATGGAGTTCCTATAGGGTCCTTTAAAATATACCATAAAAATCTGAGTGACTATCTCACAGTGTACGGACCTAAAGTTCCCCCATA‑GGGGTACCTAAAGCCCAGCCAATCACCTAAAGTCAACCTTCGGTTGACCTTGAGGGTTCACTAAGGGTTGGGGa                                                                <  1:100213/300‑1 (MQ=255)`  
                                                                         `taTTGCATCACACCAATTCATCCCTATAGAGTCAAGTCCTAAGGTATACCCATAA‑ATAGCCTCTAATGGTCTATCCTAAGGTCTATACCT‑AAAGATAGGCCATCCTATCAGTGTCACCTAAAGAGGGTCTTAGAGAGGGCCTATGGAGTTCCTATAGGGTCCTTTAAAATATACAATAAAAATCTGGGTGACTATCTCACAGTGTACGGACCTAAAGTTCCCCCATA‑GGGGTACCTAAAGCCCAGCCAATCACCTAAAGTCAACCTTCGGTTGACCTTGAGGGTTCCCTAAGGGTTGGGGa                                                                <  1:308700/300‑1 (MQ=255)`  
                                                                         `gATTGCATCACACCACTTCATCCCTATATAGTCAAGTCCTAAGGTATACCCATAA‑AGAGCCTCTAATGGTCTATCCTAAGGTCTATACCT‑AAAGATAGGCCATCCTATCAGTGTCACCTAAAGAGGGTCTTAGAGAGGGCCTATGGAGTTCCTATAGGGTCCTTTAAAATATACCATAAAAATCTGAGTGACTATCTCACAGTGTACGGACCTAAAGTTCCCCCATA‑GGGGTACCTAAAGCCCAGCCAATCACCTAAAGTCAACCTTCGGTTGACCTTGAGGGTTCCCTAAGGGTTGGGGa                                                                >  1:5737/1‑301 (MQ=255)`  
                                                                         `gATTGCATCACACCACTTCATCCCTATAGAGTCAAGTCCTAAGGTATACCCATAA‑AGAGCCTCTAATGGTCTATCCTAAGGTCTATACCT‑AAAGATAGGCCATCCTATCAGTGTCACCTAAAGAGGGTCTTAGAGAGGGCCTATGGAGTTCCTATAGTGTCCTTTAAAATATACCATAAAAATCTGAGTGACTATCTCACAGTGTACGGACCTAAAGTTCCCCCATA‑GGGGTACCTAAAGCCCAGCCAATCACCTAAAGTCAACCTTCGGTTGATCTTGAGGGTTCCCTAAGGGTTGGGGa                                                                <  1:153382/301‑1 (MQ=255)`  
                                                                         `aaTTGCATCACAACAAGTCATACATAGAGAATCAAGTCCTAAGGTAAACACATAA‑AGGGCCTCTAATGGTCTATCCTAAGGGCTATACCT‑AAAGATAGGCCATCCTATCAGTGTCACCTAAAGAGGGTATTAGAGAGTGCCTATGGAGTTCCTATAGGGTCCTTTAAAATATACCAAAAATATCTGATTGACTATCTCAAAGTGTACGGAACTAAAGTTTCCCCATA‑GTGGTACCTAAAGCCCAGCCAATCCCCTAAAGTCAACCTTCGGTTGACCTTGAGGGTTCCCTAAGGGTTGGGGa                                                                <  1:452359/300‑1 (MQ=37)`  
                                                                           `ttGCATCACACCACTTCATCCCTATAGAGTCAAGTCCTAAGGTATACTCATAA‑AGAGCCTCTAATGGTCTATCCTAAGGTCTATACCT‑AAAGATAGGCCATCCTATCAGTGTCACCTAAAGAGGGTCTTAGAGAGGGCCTATGGAGTTCCTATAGGGTCCTTTAAAATATACCATAAAAATCTGAGTGACTAACTCACAGTGTACGGACCTAAAGTTCCCCCATA‑GGGGTACCTAAAGACCAGCCAATCACCTAAAGTCAACCTTCGGTTGACCTTGAGGGTTCCCTAAGGGTTGGGGATg                                                              >  1:344941/1‑301 (MQ=255)`  
                                                                               `tataagggacatCATCTCTATAGAGTCAAGTCCTAAGGTATACCCATAA‑AGAGCCTCTAATGGTCGATCCTAAGGTCTATACCT‑AAAGATAGGCCATCCTATCAGTGTCACCTAAAGAGGGTCTTAGAGAGGTCCTATGGAGTTCCTATAGGGTCCTTTAAAATATACCATAAAAATCTGAGTGACTATCTCACAGTGTACGGACCTAAAGTTCCCCCATA‑GGGGTACCTAAAGCCCAGCCAATCACCTAAAGTCAACCTTCGGTTGACCTTGAGGGTTCCCTAAGGGTTGGGGATGAccc                                                          <  1:127401/290‑1 (MQ=255)`  
                                                                                   `caccacTTCATCCCTATAGAGTCAAGTCCTAAGGTATACCCATAA‑AGAGCCTCTAATGGTCTATCCTAAGGTCTATACCT‑AAAGATAGGCCATCCTATCAGTGTCACCTAAAGAGGGTCTTAGAGAGGGCCTATGGAGTTCCTATAGGGTCCTTTAAAATATACCATAAAAATCTGAGTGACTATCTCACAGTGTACGGACCTAAAGTTCCCCAATA‑GGGGTACCTAAAGCCCAGCCAATCACCTAAAGTCAACCTTCGGTTGACCTTGAGGGTTCCCTAAGGGTTGGGGactgtctctta                                                      >  1:171473/1‑291 (MQ=255)`  
                                                                                   `caccacTTCATCCCTATAGAGTCAAGTCCTAAGGTATACCCATAA‑AGAGCCTCTAATGGTCTATCCTAAGGTCTATACCT‑AAAGATAGGCCATCCTATCAGTGTCACCTAAAGAGGGTCTTAGAGAGGGCCTATGGAGTTCCTATAGGGTCCTTTAAAATATACCATAAAAATCTGAGTGACTATCTAACAGTGTACGGACCTAAAGTTCACCCATA‑GGGGTAACTAAAGCCCAGCCAATCACCTAAAGTCAACCTTCGGTTGACCTTGCGGGTTCCCTAAGGGTTGGGGATGACCCTTgg                                                      >  1:110687/1‑301 (MQ=255)`  
                                                                                    `cctacTTCATCCCTATAGAGTCAAGTCCTAAGGTATACCCATAA‑AGAGCCTCTAATGGTCTATCCTAAGGTCTATACCT‑AAAGATAGGCCATCCTATCAGTGTCACCTAAAGAGGGTCTTAGAGAGGGCCTATGGAGTTCCTATAGGGTCCTTTAAAATATACCATAAAAATCTGAGTGACTATCTCACAGTGTACGGACCTAAAGTTCCCCCATA‑GGGGTACCTAAAGCCCAGCCAATCACCTAAAGTCAACCTTAGGTTGACCTTGAGGGTTCCCTAAGGGTTGGGGATGACCCTTggg                                                     <  1:211507/298‑1 (MQ=255)`  
                                                                                    `accacTTTCACCCTATAGAGTCAAGTCCTAAGGTATACCCATAA‑AGAGACTCTAATGGTCTATCCTAAGGTCTATACCT‑AAAGAGAGGCCATCCTATCAGTGTAACCTAAAGAGGGTCTTAGAGAGGGCCTATGGAGTTCCTATAGGGTCCTTTAAAATACACCATAAAAATCTGAGTGACTATCTCACAGTGTACGGACCTAAAGTTCCCCCATA‑GGGGTACCTAAAGCCCAGCCAATCACCTAAAGTCAACCTTCGGTTGACCTTGAGGGTTCCCTAAGGGTTGGGGATGACCCTTggg                                                     <  1:258503/301‑1 (MQ=255)`  
                                                                                    `accacTTCATCCCTATAGAGTCAAGTCCTAAGGTATACCCATAA‑AGAGCCTCTAATGGTCTATCCTAAGGTCTATACCT‑AAAGATAGGCCATCCTATCAGTGTCACCTAAAGAGGGTCTTAGAGAGGGCCTATGGAGTTCCTATAGGGTCCTTTAAAATATACCATAAAAATCTGAGTGACTATCTCACAGTGTACGGACCTAAAGTTCCCCCATA‑GGGGTACCTAAAGCCCAGCCAATCACCTAAAGTCAACCTTCGGTTGACCTTGAGGGTTCCCTAAGGGTTGGGGATGACCCTTggg                                                     <  1:273818/301‑1 (MQ=255)`  
                                                                                      `agagcattCCATATAGAAGCAAGTCCTAAGGTATACCCATAA‑ATGGCCTCTAATGGTCTATCCTAAGGTCTATACCT‑AAAGATAGGCCATCCTATCAGTGTCACCTAAAGAGGGTCTTAGAGATGGCCTATGGAGTTCCTATAGGGTCCTTTAAAATATACCATAAAAATCTGAGTGACTATCTCACAGTGTACGGACCTAAAGTTCCCCCATA‑GGGGTACCTAAAGCCCAGCCAATCACCTAAAGTCAACCTTCGGTTGACCTTGAGGGTTCCCTAAGGGTTGGGGATGACCCTTGGGtt                                                   <  1:175026/294‑1 (MQ=255)`  
                                                                                       `acTTCATCCCTATAGAGTCAAGTCCTAAGGTATACCCATAA‑AGAGCCTCTAATGGTCTATCCTAAGGTCTATACCT‑AAAGATAGGCCATCCTATCAGTGTCACCTAAAGAGGGTCTTAGAGAGGGCCTATGGAGTTCCTATAGGGTCCTTTAAAATATACCATAAAAATCTGAGTGACTATCTCACAGTGTACGGACCTAAAGTTCTCCAATA‑GGGGTACCTAAAGCCCAGCCAATCACCTAAAGTCAACCTTCGGTTGACCTTGAGGGTTCACTAAGGGTTGGGGATGACCCTTggctgt                                                  >  1:299691/1‑297 (MQ=255)`  
                                                                                       `acTTCATCCCTATAGAGTCAAGTCCTAAGGTATACACATAA‑AGAGCCTCTAATGGTCTATCCTAAGGTCTATACCT‑AAAGATAGGCCATCCTATCAGTGTCACCTAAAGAGGGTCTTAGAGAGGGCCTATGGAGTTCCTATAGGGTCCTTTAAAATATACCATAAAAATCTGAGTGACTATCTCACAGTGTACGGACCTAAAGTTCCCCCATA‑GGGGTACCTAAAGCCCAGCCAATCACCTAAAGTCAACCTTCGGTTGACCTTAAGGGTTCCCTAAGGGTTGGGGATGAcctgtctcttt                                                  >  1:18025/1‑292 (MQ=255)`  
                                                                                        `cTTCATCCCTATAGAGTCAAGTCCTAAGGTATACCCATAA‑AGAGCCTCTAATGGTCTATCCTAAGGTCTATACCT‑AAAGATAGGCCATCCTATCAGTGTCACCTAAAGAGGGTCTTAGAGAGGGCCTATGGAGTTCCTATAGGGTCCTTTAAAATATACCATAAAAATCTGAGTGACTATCTCACAGTGTACGGACCTAAAGTTCCCCCATA‑GGGGTACCTAAAGACCAGCCAATCACCTACAGTCAACCTTCGGTTGACCTTGAGGGTTCCCTAAGGGTTGGGGATGGACCGTGGGtttt                                                 >  1:353136/1‑300 (MQ=255)`  
                                                                                          `tCATCCATATAGAGTCAAGTCCTAAGGTATACCCATAA‑AGAGCCTCTAATGGTCTATCCTAAGGTCTATACCT‑AAAGATAGGCCATCCTATCAGTGTAACCTAAAGAGGGTCTTAGAGAGGGCATATGGAGTTCCTATAGGGTCCTTTAAAATATACCATAAAAATCTGAGTGACTATCTCACAGTGTACGGACCTAAAGTTCCCCCATA‑GGGGTACCTAAAGCCCAGCCAATCACCTAAAGTCAACCTTCGGTTGACCTTGAGGGTTCCATAAGGGTTGGGGATGACCCTTGGGTTTGTc                                               <  1:330356/301‑1 (MQ=255)`  
                                                                                           `cgtgggctcggagatggttataagagacagcccATAA‑AGAGCCTCTAATAGTCTATCCTAAGGTCTATACCT‑AAAGAAAGGCCATACTATCAGTGTCACCTAAAGAGGGTCGTAGAGAGGGCCTATGGAGTTCCTATAGGGTACTTTAAAATATACCATAAAAATCTGAGTGACTATCTCACAGTGTACGGACCTAAAGTTCCCCCATA‑GGGGTACCTAAAGCCCAGCCAATCACCTAAAGTCAACCTTCGGTTGACCTTGAGGGTTCCCTAAGGGTTGGGGATGACCCTTGGGTTTGTCt                                              <  1:446045/271‑1 (MQ=25)`  
                                                                                                `tttgagagTAAAGTCCTAATGTATACCTATAACAGTG‑CGATAATGGTCTATCCTAAGGTCTATACCT‑AAAGATAGTCCATCATATCAGTGTACCCTAAAGAGGGTCTTAGAGATGGCCTATGGAGTTCCTATAGGGTCCTTTAAAATATACCATAAAAATCTGAGTGACTATCTCACAGTGTACGGACCTAAAGTTCCCCCATA‑GGGGTACCTAAAGCCCAGCCAATCACCTAAAGTCAACCTTCGGTTGACCTTGAGGGTTCCCTAAGGGTTGGGGATGACCCTTGGGTTTGTCTTTggg                                         <  1:113321/297‑1 (MQ=25)`  
                                                                                                `tatgagagacaggtatagaGGTATACCCATAA‑TGATCCTCTAATGGTTGATCCTAAGATCTATACCG‑AAAGATAGGGCATCCTATCAGTTTCACCTAAAGAGGGTCTTAGAGAGAGCCTATGGAGTGCCAATAGGGTCCTTTAAAAGTTACCATAAAAATCTGAGTGACTATCTCACAGTGTACGGACCTAAAGTTCCCCCATA‑GGGGTACCTAAAGCCCAGCAAATCACCTAACGTCTACTTTCGGTTGACCTTGAGGGTTCCCTAAGGGTTGGGGATGACCCTTTGGTTTGTCTTTggg                                         <  1:284752/283‑1 (MQ=21)`  
                                                                                                `cTATAGATTCAAGGCCTAAGGTATACCCATAA‑AGAGCCTATAATGGTCTATCCGAAGGTCTATACCT‑AAAGATAGGCCATCCTATCAGTGTCACCTAAAGAGGGGCTTAGAGAGGGCCTATGGAGTTCCTATAGGGTCCTTGAAAATATACCATAAAAATCTGAGTGACTATCTCACAGTGTACGGACCTAAAGTTCCCCCATA‑GGGGTACCTAAAGCCCAGCCAATCACCTAAAGTCAACCGTCGGTTGACCTTGAGGGTTCCCTAAGGGTTGGGGATGACCCTTGGGTTTGTCTTTggg                                         <  1:48333/301‑1 (MQ=255)`  
                                                                                                `cTATAGAGTCAAGTCCTAAGGTATACCAATAA‑AGAGCCTCTAATGGTCTATCCTAAGGTCTATACCT‑AAAGATAGGCCATCCTATCAGTGTCACCTAAAGAGGGTATTAGAGAGTGCCTATGGAGTTCCTATAGGGTACTTTAACATATACCATAAAAATCTGAGTGACTATCTCACAGTTTACGGACCTAAAGTTCCCCCATA‑GGGGTACCTAAAGCCCAGCCAATCACCTCAAGTCAACCTTCGGTTGACCTTGAGGTTTCCCTAAGGGTTGTGGATGACCCTTGGGTGTGTCTTTggg                                         >  1:349107/1‑301 (MQ=255)`  
                                                                                                      `agatgtgtagaagagacagcccATAA‑AGAGCCTCTAATGGTCTATCCTAAGGTCTATACCT‑AAAGATAGGCCATCCGATCAGTGTCACCTAAAGAGGGTCTTAGAGAGGGCCTATGGAGTTCCTATAGGGTCCTTTAAAATATACCATAAAAATCTGAGTGACTATCTCACAGTGTACGGACCTAAATTTCCCCCATA‑GGGGTACCTAAAGCCCAGCCAATCACCTAAAGTCAACCTTCGGTTGACCTTGAGGGTTCCCTAAGGGTTGGGGATGACCCTTGGGTTTGTCTTTGGGTGTTAc                                   <  1:108023/282‑1 (MQ=255)`  
                                                                                                      `agTCAAGTCCTAAGGTATACCCATAA‑AGATCCTCTAATGGTCTATCCTAAGGTCTATACCT‑AAAGATAGGCCATCCTATCAGTGTCACCTAAAGAGGGTCTTAGAGAGGGCCTATGGAGTTCCTATAGGGTCCTTTAAAATATACCATAAAAATCTGAGTGACTATATCACAGTGTACGGACCTAAAGTTCCCCCATA‑GGGGTACCTAAAGCCCAGCCAATCACCTAAAGTCAACCTTCGGTTGACCTTGAGGGTTCCCTAAGGGTTGGGGATGACCCTTGGGTTTGTCTTTGGGTGTTAc                                   <  1:60856/301‑1 (MQ=255)`  
                                                                                                       `gTCAAGTCCTAAGGTATACCCATAA‑AGAGCCTCTAATGGTCTATCCTAAGGTCTATACCT‑AAAGATAGGCCATCCTATCAGTGTCACCTAAAGAGGGTCTTAGAGAGGGCCTATGGAGTTCCTATAGGGTCCTTTAAAATATACCATAAAAATCTGAGTGACTATCTCACAGTGTACGGACCTAAAGTTCCCCCATA‑GGGGTACCTAAAGCCCAGCCAATCACCTAAAGTCAACCTTCGGTTGACCTTGAGGGTTCCCTAAGGGTTGTGGATGACCCTTGGGTTTGTCTTTGGGTGTTAcc                                  <  1:263657/301‑1 (MQ=255)`  
                                                                                                        `tCAAGTCCTAAGGTATACCCATAA‑AGAGCCTCTAATGGTCTATCCTAAGGTCTATACCT‑AAAGATAGGCCATCCTATCAGTGTCACCTAAAGAGGGTCTTAGAGAGGGCCTATGGAGTTCCTATAGGGTCCTTTAAAATATACCATAAAAATCTGAGTGACTATCTCACAGTGTACGGACCTAAAGTTCCCCCATA‑GGGGTACCTAAAGCCCAGCCAATCACCTAAAGTCAACCTTCGGTTGACCTTGAGGGTTCCCTAAGGGTTGGGGATGACCCTTGGGTTTGTCTTTGGGTGTTACCt                                 <  1:13590/301‑1 (MQ=255)`  
                                                                                                        `cagaGTCCTAAGGTATACCCATAA‑AGAGCCTCTAATGGTCTATCCTAAGGTCTATACCT‑AAAGATAGGCCATCCTATCAGTGTCACCTAAAGAGGGTCTTAGAGAGGGCCTATGGAGTTCCTATAGGGTCCTTTAAAATATACCATAAAAATCTGAGTGACTATCTCACAGTGTACGGACCTAAAGTTCCCCCATA‑GGGGTACCTAAAGCCCAGCCAATCACCTAAAGTCAACCTTCGGTTGACCTTGAGGGTTCCCTAAGGGTTGGGGATGACACTTGGGTTTGTCTTTGGGTGTTACCt                                 <  1:472913/298‑1 (MQ=255)`  
                                                                                                        `acaggTCCTAAGGTATACCCATAA‑AGAGCCTCTAATGGTCTATCCTAAGGTATATACCT‑AAAGATAGGCCATCCTATCAGTGTCACATAAAGAGGGTCTTAGAGAGGGCCTATGGAGTTCCTATAGGGTCCTTTAAAATATACCATAAAAATCTGAGTGACTAGCTCACAGTGTACGGACCTAAAGTTCCCCCATA‑GGGGTACCTAAAGCCCAGCCAATCACCTAAAGTCAACCTTCGGTTGACCTTGAGGGTTCCCTAAGGGTTGGGGATGACCCTTGGGTTTGTCTTTGGGTGTTACCt                                 <  1:239028/297‑1 (MQ=255)`  
                                                                                                          `aaGTCCTAAGGTATACCCATAA‑AGAGCCTCTAATGGTCTATCCTAAGGTCTATACCT‑AAAGATAGGCCATCCTATCAGTGTCACCTAAAGAGGGTCTTAGAGAGGGCCTATGGAGGTCCTATAGGGTCCTTTAAAATATACCAGAAAAATCTGAGTGACTATCTAACAGTGTACGGACCTAAAGTTCCCCCATA‑GGGGTACCTAAAGCCCAGCCAATCACCTAAAGTCAACCTTCGGTTGACCTTGAGGGTTCCCTAAGGGTTGGGGATGACCCTTGGGTTTGTCTTTGGGTGTTACCTTg                               <  1:2440/301‑1 (MQ=255)`  
                                                                                                            `gTCCTAAGGTATATCCATAA‑AGAGCCTCTAATGGTCTATCCTAAGGTCTATACCT‑AAAGATAGGCCATCCTATCAGTGTCACCTAAAGAGGGTCTTAGAGAGGGCCTATGGAGTTCCTATAGGGTCCTTTAAAATATACCATAAAAATCTGAGTGACTATCTCACAGTGTACGGACCTAAAGTTCCCCCATA‑GGGGTACCTAAAGCCCAGCCAATCACCTAAAGTCAACCTTCGGTTGACCTTGAGGGTTCCCTAAGGGTTGGGGATGACCCTTGGGTTTGTCTTTGGGTGTTACCTTGAg                             >  1:391523/1‑301 (MQ=255)`  
                                                                                                            `gTCCTAAGGTATACCCATAA‑AGAGCCTCTAATGGTCTATCCTAAGGTCTATACCT‑AAAGATAGGCCATCCTATCAGTGTCACCTAAAGAGGGTCTTAGAGAGGTCCTATGGAGTTCCTATAGGGTCCTTTAAAATATACCATAAAAATCTGAGTGACTATCTCACAGTGTACGGACCTAAAGTTCCCCCATA‑GGGGTACCTAAAGCCCAGCCAATCACCTAAAGTCAACCTTCGGTTGACCTTGAGGGTTCCCTAAGGGTTGGGGATGACCCTTGGGTTTGTCTTTGGGTGTTACCTTGAg                             <  1:456378/301‑1 (MQ=255)`  
                                                                                                              `cccaaGGGATAACCAAAA‑AGAACCTCTTATTGGTTATCCTAAGGTCTATACCT‑AAAGATAGGCCATCCTATCAGTGTCACCTAAAGAGGGTCTTAGAGAGGGCCTATGGAGTTCCTATAGGGTCCTTTAAAATATACCATAAAAATCTGAGTGACTATCTCACAGTGTACGGACCTAAAGTTCCCCCATA‑GGGGTACCTAAAGCCCAGCCAATCACCTAAAGTCAACCTTCGGTTGACCTTGAGGGTTCCCTAAGGGTTGGGGATGACCCTTGGGTTTGTCTTTGGGTGTTACCTTGAgtg                           <  1:139869/298‑1 (MQ=25)`  
                                                                                                              `ccTAAGGTATACCCATAA‑AGAGCCTATAATGGTCTATCCTAAGGTCAATACCT‑AAAGATAGGCCATCCTGTCAGTGTAAACTAAAGAGTGTCGTAGAGAGGGCATATGGAGTTCCTATAGGGTCCTTTAAAATATACCATAAAAATCTGAGTGACTATCTCACAGTGTACGGAGCTAAAGTGCCCCCATA‑GGTGTACCTAAAGCCCAGCCAATCTCCTAAAGTCAACCTTCGGTTGACCTTGAGGGTTCCCTAAGGGTTGAGGATGACCCTTGGGTTTGTCTGTGGGTGTTACCTTGAgtg                           <  1:84835/301‑1 (MQ=25)`  
                                                                                                              `ccTAAGCTATACCCATAA‑AGAGCCTCTAATTGTCTATCCTAAGGTCTATACCT‑AAAGATAGGCCATCCTATCAGTGTCACCTCAAGAGGGTCTTAGATAGGGCCTATGCAGTTCCTATAGGGTCCTTTAAAATATACCATAAAAATCTGAGTGACTATCTCACAGTGTACGGACCTAAAGTACCCCCATA‑GGGGTACCTAAAGCCCAGCCAATCACCTAAAGTCAACCTTCGGTTGGCCTTGAGGGTTGCCTAAGGTTTGGGGATGACCCTTGGGTTTGTCTTTGGGTGTTACCTTGAgtg                           <  1:433764/301‑1 (MQ=37)`  
                                                                                                              `attagagccagcccAGAA‑ATATCCGCTAATGGTCTATCCTACAGTCTATACCT‑AAAGATACGCTATCCTATCAGTGTCACCTAAAGAGGGTCTTAGGGAAGGCCTATGGAGTTCCTATAGGGTCCTTTAAAATATACCATAAAAATATGAGTGACTATCTCACAGGGGACGGACATAAAGTTCCCCCATA‑GGGGTACCTAAAGCCCAGCCAATCACCTAAAGTCAACTTTCGGTTGACCTTGAGGGTTCCCGAAAGGTTGGGGATGACCCTTGGGTTTGTCTTTGGGTGTTACCTTGAgtg                           <  1:251492/290‑1 (MQ=21)`  
                                                                                                              `aagaaGGTATAGCAATAA‑AGGGCGTCAAAGGGTCAATGTAAAGGTCTAAACCT‑AAAGATAGGCAATCCTATCAGTGTGAACTAAAGAGGGTCTTAGATAGGGCCTATGGAGTTCCTATAGGGTACTTTAAAATATACCATAAAAATCTGAGTGACTATCTCAAAGGGTACGGACATAAAGTT‑CCCCATA‑GGGGTACCTAAAGCCCAGCCAGTCACCTAAAGTCAACCTTCGGTTGACCTTGAGGGTTCCCTAAGGGTTGGGGATGACCCTTGGGTTTGTCTTTGGGTGTTACCTTGAgtgt                          <  1:166699/298‑1 (MQ=21)`  
                                                                                                              `aaacgggtaataccAAAA‑AAAGCCTCTAAAGGTATATCCTAAGGGCTAGAACT‑AAAGATAGGACCTTCTATCAGTGTCACCTAAAGAGGGTCTTAGAGAGGGCCTATGGAGTTCCTATAGGGTCCTTTAAAATATACCATAAAAATCTGAGTGACTATCTCACAGTGTACGGACCTAAAGTTCCCCCATA‑GGGGTACCTAAAGCCCAGCCAATCACCTAAAGTCAACCTTCGGTTGACCTTGAGGGTTCCCTAAGGGTTGGGGATGACCCTTGGGTTTGTCTTTGGGTGTTACCTTGAgtg                           <  1:479301/289‑1 (MQ=21)`  
                                                                                                               `cTAAGGTATACCCATAA‑AGAGCCTCTAATGGTCTATCCTAAGGTCTATACCT‑AAAGATAGGCCATCCTATCAGTGTCACCTAAAGAGGGTCTTAGAGAGGGCCTATGGAGTTCCTATAGGGTCCTTTAAAATATACCATAAAAATCTGAGTGACTATCTCACAGTGTACGGACCTAAAGTTCCCCCATA‑GGGGTACCTAAAGCCCAGCCAATCACCTAAAGTCAACCTTCGGTTGACCTTGAGGGTTCCCTAAGGGTTGGGGATGACCCTTGGGTTTGTCTTTGGGTGTTACCTTGAgtgt                          <  1:414488/301‑1 (MQ=255)`  
                                                                                                                  `ggtacagcccATAA‑ATAGCCTCTAATTGTCTATCCTAGTGTCTATACCT‑AAAGATAGGCCATCCTATCAGTGTCACCTAAAGAGGGTCTTAGAGAGGGCCTATGGAGTTCCTATAGGGTCCTTTAAAATATACCATAAAAATCTGAGTGACTATCTCACAGTGCACGGACCTAAAGTTCCCCCATA‑GGGGTACCTAAAGCCCAGCCAATCACCTAAAGTCAACCTTCGGTTGACCTTGAGGGTTCCCTAAGGGTTGGGGATGACCCTTGGGTTTGTCTTTGGGTGTTACCTTGAGTGtctc                       <  1:231072/294‑1 (MQ=255)`  
                                                                                                                  `gggTATACCCATAA‑AGAGCCTCTAATGGTCTATCCTAAGGTCTATACCT‑AAAGATAGGCCATCCTATCAGTGTCACCTAAAGAGGGTCTTAGAGAGGGCCTATGGAGTTCCTATAGGGTCCTTTAAAATATACCATAAAAATATGAGTGACTATCTCACAGTGTACGGACCTAAAGTTCCCCCATA‑GGGGTACCTAAAGCCCAGCCAATCACCTAAAGTCAACCTTCGGTTGACCTTGAGGGTTCCCTAAGGGTTGGGGATGACCCTTGGGTTTGTCTTTGGGTGTTACCTTGAGTGtctc                       <  1:444115/300‑1 (MQ=255)`  
                                                                                                                    `gacagcccATAA‑AGAGCCTCTAATGTGCTATCCTAAGGTCTATACCT‑AAAGATAGGCCATCCTATCAGTGTCACCTAAAGAGGGTCTTAGAGAGGGCCTATGGAGTTCCTATAGGGTCCTTTAAAATATACCATAAAAATCTGAGTGACTATCTCACAGTGTACGGACCTAAAGTTCCCCCATA‑GGGGTACCTAAAGCCCAGCCAATCACCTAAAGTCAACCTTCGGTTGACCTTGATGGTACCCTAAGGGTTGGGGATGACCCTTGGGTTTGTCTTTGGGTGTTACCTTGAGTGtctctc                     <  1:70083/296‑1 (MQ=255)`  
                                                                                                                    `gTATACCCATAA‑AGAGCCTCTAATGGTCTATTCTAAGGTCTATCCCT‑AAAGATAGGCCATCCTATCAGTGTCAACTAAATAGGGTCTTAGAGAGGGCCGATGGAGTTCCTATAGGGTCCTTTAAAATATACCATAAAAATCTGAGTGACTATCTCACAGTGTACGGACCTAAAGTTCCCCCATA‑GTGGTACCTAAAGCCCAGCCAATCACCTAAAGTCAACCTTCGGTTGACCTTGAGGGTTCCCTAAGGGTTGGGGATGACCCTTGGGTTTGTCTTTGGGTGTTACCTTGAGTGtctctc                     <  1:374050/301‑1 (MQ=255)`  
                                                                                                                     `ttgtgtctcagagacagctctACTGTTCTATCCTAAGGTCTATACCT‑AAAGATCGGCCATCCTATCAGTGTCTCCTAAATAGGTTCTTAGAGAGGGCCTATGGAGTTCCTATATGGTCCTTTACAATATACCATAAAATTCTGAGTGACTATCTCACAGTGTACGGACCTAAAGTTCCCCCATA‑GGGGTACCTAAAGCCCAGCCAATCACCTAAAGTCAACCTTCGGTTGACCTTGAGGGTTCCCTAAGGGTTGGGGATGACCCGTGGGTTTGTCTTTGGGTGTTACCTTGAGTGtctctc                     <  1:326388/284‑1 (MQ=21)`  
                                                                                                                     `ttgtataagagacaggCCTCTAATGGTCTATCCTAAGGTCTATACCT‑AAAGATAGGCCATCCTATCAGTGTCACCTAAAGAGGGTCTTAGAGAGGGCCTATGGAGTTCCTATAGGGTCCTTTAAAATATACCATAAAAATCTGAGTGACTATCTCACAGTGTACGGACCTAAAGTTCCCCCATA‑GGGGTACCTAAAGCCCAGCCAATCACCTAAAGTCAACCTTCGGTTGACCTTGAGGGTTCCCTAAGGGTTGGGGATGACCCTTGGGTTTGTCTTTGGGTGTTACCTTGAGTGtctctc                     <  1:407740/286‑1 (MQ=255)`  
                                                                                                                     `tataCCCATAA‑AGAGCCTCTAATGGTCTATCCGAAGGTCTATACCT‑AAAGATAGGCCATCCTAGCAGTGTCACCTAAAGAGGGTCTTAGAGAGGGCCTATGGAGTTCCTATAGGGTCCTTTAAAATATACCATAAAAATCGGAGTGACTATCTCACAGTGTACGGACCTAAAGTTCCCCCATAGGGGGTACCTAAAGCCCAGCCAATCACCTAAAGTCAACCTTCGGTTGACCTTGAGGGTTCCCTAAGGGTTGGGGATGACCCTTGGGTTTGTCTTTGGGTGTTACCTTGAGTGtctctc                     <  1:18514/301‑1 (MQ=255)`  
                                                                                                                     `gtgggctcggagatgtgtataagagacagaTCCTAAGGTCTATACCT‑AAAGATAGGCCATCCTATCAGTGTCACCTAAAGAGGGTCTTAGAGAGGGCCTATGGAGTTCCTATAGGGTCCGTTAAAATATACCATAAAAATCTGAGTGACTATCTCACAGTGTATGGACCTAAAGTTCCCCCATA‑GGGGTACCTAAAGCCCAGCCAATCACCTAAAGTCAACCTTCGGTTGACCTTGAGGGTTCCCTAAGGGTTGGGGATTACCCTTGGGTTTGTCTTTGGGTGTTACCTTGAGTGtctctc                     <  1:229042/272‑1 (MQ=25)`  
                                                                                                                      `ataGCCATAA‑AGAGCCTCTAATGGTCTATCCTAAGGTCTATACCT‑AAAGATAGGCCATCCTATCAGTGTCAACTAAAGAGGGTCTTAGAGAGGGCCTATGGAGTTCCTATAGGATCCTTTAAAATATACCATAAAAAGCTGAGTGACTATCTCACAGTGTACGGACCTAAAGTTCCCCCATA‑GGGGTACCTAAAGCCCAGCCAATCGCCTAAAGTCAACCTTCGGATGACCTTGAGGGTTCCCTAAGGGTTGGGGATGACCCTTGGGTTTGTCTTAGGGTGTTACCTTGAGTGTCTCTCtg                   <  1:124459/301‑1 (MQ=255)`  
                                                                                                                       `gggcgaggagaagtgtataagagacagagacTAAGGTATATAACT‑AAAGATAGGCCATCCTATCAGTGTAACCAAAAAAGGGTCATAGAGAGGGGCAATGGAGTTACTATAGGGGCCGTTAAAATATAACAAAAAAATCGGAGTGACTATCTCAAAGTGAACGGACCAAAAGTACCCCCATA‑GGGGTACCTAAAGGACAGAAAATCAACTAAAGTCAACAGTCGGTTGACATTGAGGGTTCCCTAAGGGTTGGGGATGACCCTTGGGTTTGTCTTTGGGTGTTACCTTGAGTGTCTCTCtg                   <  1:209656/271‑1 (MQ=18)`  
                                                                                                                         `cccATAA‑AGAGCCTCTAATGGTCTATCCTAAGGTCTATACCT‑AAAGATAGGCCATCCTTTCAGTGTCACCTAAAGAGGGTCTTAGAGAGGGCCTATGGAGTTCCTATAGGGTCCTTTAAAATATACCATAAAAATCTGAGTGACTATCTCACAGTGTACGGACCTAAAGTTCCCCCATA‑GGGGTACCTAAAGCCCAGCCAATCACCTAAAGTCAACCTTCGGTTGACCTTGAGGGTTCCCTAAGGGTTGGGGATGACCCTTGGGTTTGTCTTTGGGTGTTACATTTAGTGtctcctgtctc                >  1:425303/1‑294 (MQ=255)`  
                                                                                                                         `cccATAA‑AGAGCCTCTAATGGTCTATCCTAAGGTCTATACCT‑AAAGATAGGCCATCCTATCAGTGTCACCTAAAGAGGGTCTTAGAGAGGGCCTATGGAGTTCCTATAGGGTCCTTTAAAATATACCATAAAAATCTGAGTGACTATCTCACAGTGTACGGACCTAAAGTTCCCCCATA‑GGGGTACCTAAAGCCCAGCCAATCACCTAAAGTCAACCTTCGGTTGACCTTGAGGGTTCCCTAAGGGTTGGGGATGACCCTTGGGTTTGTCTCTgtctcttatacacatctccgagcccacg                >  1:437773/1‑274 (MQ=37)`  
                                                                                                                         `cccATAA‑AGAGCCTCTAATGGTCTATCCTAAGGTCTATACCT‑AAAGATAGGCCATCCTATCAGTGTCACCTAAAGAGGGTCTTAGAGAGGGCCTATGGAGTTCCTATAGGGTCCTTTAAAATATACCATAAAAATCTGAGTGACTATCTCACAGTGTACGGACCTAAAGTTCCCCCATA‑GGGGTACCTAAAGCCCAGCCAATCACCTAAAGTCAACCTTCGGTTGACCTTGAGGGTTCCCTAAGGGTTGGGGATGACCCTTGGGTTTGTCCTTGGGTGTTACCTTGAGTGtcgcctgtctt                >  1:200259/1‑292 (MQ=255)`  
                                                                                                                         `cccATAA‑AGAGCCTCTAATGGTCTATCCTAAGGTCTATACCT‑AAAGATAGGCCATCCTATCAGTGTCACCTAAAGAGGGTCTTAGAGAGGGCCTATGGAGTTCCTATAGGGTCCTTTAAAATATACCATAAAAATCTGAGTGACTATCTCACAGTGTACGGACCTAAAGTTCCCCCATA‑GGGGTACCTAAAGCCCAGCCAATCACCTAAAGTCAACCTTCGGTTGACCTTCAGGGTTCCCTAAGGGTTGGGGATGACCCTTGGGTTTGTCTTTGGGTGTTACCtctgtctcttatccactt                >  1:121161/1‑284 (MQ=255)`  
                                                                                                                                      `ctctAATGGTCTATCCTAAGGTCTATACCT‑AAAGATAGGCCATCCTATCAGTGTCACCTAAAGAGGGTCTTAGAGAGGGCCTATGGAGTTCCTATAGGGTCCTTTAAAATATACCATAAAAATCTGAGTGACTATCTCACAGTGTACGGACCTAAAGTTCCCCCATA‑GGTGTACCTAAAGCCCAGCCAATCACCTAAAGTCAACCTTCGGTTGACCTTGAGGGTTCCCTAAGGGTTGGGGATGACCCTTGGGTTTGTCTTTGGGTGTTACCtctgtctcttatacacatctccgagcccac    >  1:506665/1‑272 (MQ=25)`  
                                                                                                                                        `ctAATGGTCTATCCTAAGGTCTATACCT‑AAAGATAGGCCATCCTGTCAGTGTCACCTAAAGAGGGTCTTAGAGAGGGCCTATGGAGTTCCTATAGGGTCCTTTAAAATATACCATAAAAATCTGAGTGACTATCTCACAGTGTACGTACCTAAAGTTCCCCCATA‑GGGGTACCTAAAGCCCAGCCAATCACCTAAAGTCAACCTTCGGTTGACCTTGAGGGTTCCCTAAGGGTTTGGGATGACCCTTGGGTTTGTCTTTGGGTGTTACCTTGAGTGTCTCTCtgctgtctcttatacacat  >  1:337439/1‑284 (MQ=25)`  
                                                                                                                                        `ctAATGGTCTATCCTAAGGTCTATACCT‑AAAGATAGGCCATCCTATCAGTGTCACCTAAAGAGGGTCTTAGAGAGGGCCTATGGAGTTCCTATAGGGTCCTTTAAAATATACCATAAAAATCTGAGTGACTATCTCACAGTGTACGGACCTAAAGTTCCCCCATA‑GGGGTACCTAAAGCCCAGCCAATCACCTAAAGTCAACCTTCGGTTGACCTTGAGGGTTCCCTAAGGGTTGGGGATGACCCTTGGGTTTGTCTTTGGGTGTTCCCTTGAGTGTCTCTCtgctgtctcttatacacat  >  1:216299/1‑284 (MQ=255)`  

                                                                                                                                                                                                                                                                                                              `|`                                                                                                                                          
`TTGCTCTATACTCACTTGTAGCGATTAGGGTCTTCCTGACCGACTGATGGCTCACCGAGGGATTCAGCGGTATGATTGCATCACACCACTTCATCCCTATAGAGTCAAGTCCTAAGGTATACCCATAA‑AGAGCCTCTAATGGTCTATCCTAAGGTCTATACCT‑AAAGATAGGCCATCCTATCAGTGTCACCTAAAGAGGGTCTTAGAGAGGGCCTATGGAGTTCCTATAGGGTCCTTTAAAATATACCATAAAAATCTGAGTGACTATCTCACAGTGTACGGACCTAAAGTTCCCCCATAGGGGGTACCTAAAGCCCAGCCAATCACCTAAAGTCAACCTTCGGTTGACCTTGAGGGTTCCCTAAGGGTTGGGGATGACCCTTGGGTTTGTCTTTGGGTGTTACCTTGAGTGTCTCTCTGTGTCCCT‑‑‑‑‑‑‑‑‑‑  >  NC_001604.1/39511‑39937`

  

Alignment Legend

Aligned base mismatch/match (shaded by quality score): `ATCG/ATCG` `< 3 ≤` `ATCG/ATCG` `< 10 ≤` `ATCG/ATCG` `< 18 ≤` `ATCG/ATCG` `< 33 ≤` `ATCG/ATCG` `< 38 ≤` `ATCG/ATCG`

Unaligned base: `atcg`    Masked matching base: `atcg`    Alignment gap: `‑`    Deleted base: `‑`
